# Effects of Congruent and Incongruent Stimulus Colour on Flavour Discriminations

**DOI:** 10.1177/2041669518761463

**Published:** 2018-03-09

**Authors:** Leonie Wieneke, Pauline Schmuck, Julia Zacher, Mark W. Greenlee, Tina Plank

**Affiliations:** Institute for Experimental Psychology, University of Regensburg, Germany; Institute for Psychology, University of Bonn, Germany; Institute for Experimental Psychology, University of Regensburg, Germany

**Keywords:** colour perception, flavour perception, multisensory integration

## Abstract

In addition to gustatory, olfactory and somatosensory input, visual information plays a role in our experience of food and drink. We asked whether colour in this context has an effect at the perceptual level via multisensory integration or if higher level cognitive factors are involved. Using an articulatory suppression task, comparable to Stevenson and Oaten, cognitive processes should be interrupted during a flavour discriminatory task, so that any residual colour effects would be traceable to low-level integration. Subjects judged in a three-alternative forced-choice paradigm the presence of a different flavour (triangle test). On each trial, they tasted three liquids from identical glasses, with one of them containing a different flavour. The substances were congruent in colour and flavour, incongruent or uncoloured. Subjects who performed the articulatory suppression task responded faster and made fewer errors. The findings suggest a role for higher level cognitive processing in the effect of colour on flavour judgements.

## Introduction

The effects that colours have on the human experience while tasting food and beverages are well known. Many studies have already discovered that the congruence between colour and taste or smell and flavour not only affects the perceived intensity of the food but also has an effect on how difficult it is to identify the flavour or odour (e.g. Morrot, Brochet, & Dubourdieu, 2001; Zellner, Bartoli, & Eckard, 1991; Zellner & Whitten, 1999). For example, the perceived sweetness of cherry-flavoured solutions can be enhanced by adding red colour (Johnson & Clydesdale, 1982; Lavin & Lawless, 1998). The more intense the colour, the sweeter the taste. This is usually observed when a colour is naturally associated with a certain flavour. Cherries, for example, are associated with red, whereas lemons are associated with yellow. When a colour is paired with a congruent flavour, identification of the flavour is facilitated. This also works the other way around – if you mix cherry flavour with incongruent yellow food colouring, subjects find it harder to identify the flavour and make more errors. DuBose, Cardello, and Maller (1980), for example, showed that incongruently coloured beverages were identified significantly less often than were congruently coloured ones. These misidentifications also occur when the subjects are explicitly alerted to the fact that flavour and colour can be independent of each other (e.g. Stillman, 1993; Zampini, Sanabria, Phillips, & Spence, 2007).

Although many studies showed that these colour effects exist, it remains unclear how exactly colour affects olfaction or gustatory perception. The two main approaches here differ concerning the assumed processing level at which colours moderate the flavour experience. One theory expects low-level processes in terms of multisensory integration on an early perceptual stage (bottom-up approach). Such an effect could occur, if sensory information from the visual domain (in this case colour) and olfactory or gustatory stimuli are integrated into a multisensory percept, before further cognitive processes could influence the identification (Spence et al., 2010). Österbauer et al. (2005), for example, showed that congruent colour–odour pairs can automatically activate the secondary olfactory cortex. The second approach concentrates on a cognitive process. This can be briefly described as top-down processes that modulate the flavour percept while eating or drinking. Colour manipulates the expectancies people have with respect to perceived taste or smell (Spence et al., 2010), expectancies that build up by learning colour–flavour associations (Shankar, Levitan, Prescott, & Spence, 2009), as they naturally occur.

Stevenson and Oaten (2008) isolated perceptual from cognitive influences of colours on olfactory discrimination. They investigated the phenomenon with a triangle test method. On each trial, subjects were given three cups with liquids they had to discriminate according to their smell. Two of the cups contained the same odour, and the third cup contained a different odour. The different one had to be selected. On each trial, the three cups contained liquids with similar odours, for example, strawberry and cherry, or lemon and grapefruit. In addition, the colours of the liquids were varied. The liquids could have the appropriate colour (e.g. red for strawberry/cherry trials), the inappropriate colour (e.g. yellow for strawberry/cherry trials) or could be colourless. Stevenson and Oaten hypothesised that, when the colour directly activated a certain odour representation, both the appropriate colours and the inappropriate colours should impair odour discrimination relative to the colourless condition, because the representation of the uniform colour should hamper both. On the other hand, should colour assist identification, it could facilitate discrimination in the colour-appropriate condition and disturb discrimination in the colour-inappropriate condition. Their results showed that the participants performed worse in the colour-inappropriate condition, indicating an expectation effect on odour discrimination with appropriate colours assisting identification in this experimental set-up.

In a second experiment, Stevenson and Oaten (2008) additionally implemented an articulatory suppression task (AST) that the participants had to perform during the interstimulus intervals and during the decision process. Articulatory suppression is used to disrupt cognitive processes by performing a distracting verbal task (in this case, the aloud repetition of the word ‘the’). According to Landry and Bartling (2011), the AST can be used to disrupt processes in the phonological loop of working memory (Baddeley, 2003). Stevenson, Sundqvist, and Mahmut (2007) had already used the AST in an odour-only triangle test to prevent participants from naming the odours they had sniffed. Thus, the AST was used to force their participants to base their decisions on a perceptual encoding of the odours instead of relying on the verbal labels that participants possibly assigned to the odours and rehearsed in the phonological loop of their working memory until decision. Similarly, in Stevenson and Oaten’s (2008) second experiment, this distracting verbal task was expected to disturb verbal labelling based on colour-induced expectancy effects. As Zellner et al. (1991) pointed out, colours could activate a specific set of verbal labels for an odour based on former experiences, thereby enhancing odour identification. Assuming that discrimination is mediated by semantic processes, Stevenson and Oaten (2008) hypothesised that the AST should hamper odour identification and any form of labelling based on the colour participants see before sniffing, thereby improving performance in the inappropriate-colour condition. Their results indicate that participants performed better in the incongruent-colour condition with the AST.

In the current study, we largely aimed to replicate the experiments of Stevenson and Oaten (2008) in the flavour domain and additionally collected response times to further investigate the underlying effects. We also applied a triangle test method, presenting three liquids on each trial, of which one had a different flavour that had to be singled out. The liquids were colourless or were coloured congruently or incongruently with respect to their flavours. Half of the participants had to perform an AST during the tasting, and the other half had no such task. We expected – in line with Stevenson and Oaten – that the incongruent condition would be more difficult, resulting in more discrimination errors and longer response times, especially in the group without the AST. This hypothesis is based on the following assumption: If colour assists flavour identification by activating a certain set of flavours, and flavour identification assists discrimination in the triangle test, then performance in the incongruent colour condition should be worse, because the ‘wrong’ set of possible flavours would be activated during tasting. Also, we expected the AST to disrupt cognitive processes during the discrimination task, leading to an overall better and faster discrimination process in the AST group compared to the non-AST group. If the AST group does not show better performance, colour effects could, at least partially, be attributed to low-level multisensory integration.

## Methods

### Participants

Ninety participants (20 males and 70 females) took part in this experiment. They were between 18 and 65 years (mean age = 28.6; *SD* = 12.7). All participants reported themselves to have normal or corrected-to-normal visual acuity and normal colour vision, additionally screened for colour-vision deficiencies by the use of Ishihara plates in 60 subjects (Ishihara, 1968), and were non-smokers. None of the participants had a history of medical treatment in the nasal cavity or throat that could have impaired gustatory or olfactory perception. The experimental procedures and consent forms were approved by the Ethics Commission of the University of Regensburg. Participants gave written informed consent and were randomly assigned to one of two experimental groups (AST group: *n* = 45; non-AST group: *n* = 45).

### Materials

**Figure 1.**
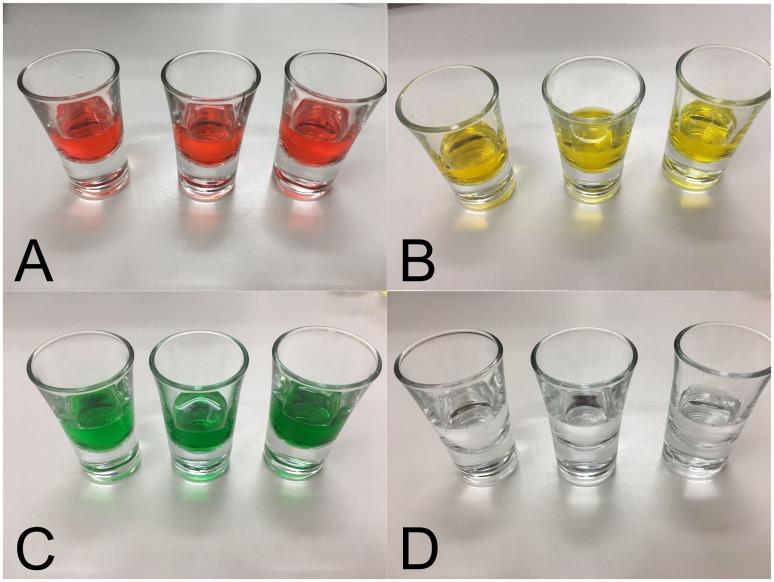
Examples for the colour conditions in this experiment: (a) red colouring, (b) yellow colouring, (c) green colouring and (d) colourless condition.

Four flavours (strawberry, cherry, lemon and grapefruit from Elli’s Aromen, https://www.ellisaromen.de) were used. Three drops of each concentrate were mixed with 500 ml of water and then presented to the participants in portions of 10 ml each in shot glasses to avoid any distortion of flavours by plastic cups. Comparable to the task used by Stevenson and Oaten (2008), the flavours were presented in a triangle test method, while always the flavours strawberry and cherry or lemon and grapefruit were combined in one trial. These combinations were used, because they are quite similar and thus hard to distinguish. Also, the added flavours were of low concentration. The flavour solutions were presented colourless (no food colouring added), in congruent colour–flavour combinations (red food colouring for strawberry/cherry trials; yellow food colouring for lemon/grapefruit trials) or in incongruent colour–flavour combinations (yellow food colouring for strawberry/cherry trials; green food colouring for lemon/grapefruit trials) (see Figure 1). For food colouring, the colours ‘red’, ‘golden yellow’ and ‘leaf green’ (Wilton) were used. In a separate control test, six subjects were asked to discriminate the coloured and uncoloured liquids without any flavour added in a triangle test blindfolded. All six subjects in the control test indicated that they could neither smell nor taste any differences between the coloured and uncoloured liquids, and the performance in this control triangle test was at chance level. Participants in the main experiment were not informed about the flavours used in the experiment and also not explicitly alerted to the colours, but the colours of the solutions were clearly visible.

### Procedure

The experiment was conducted by three of the authors (LW, PS and JZ) at five different locations, but the procedure was always identical. The experiment consisted of 24 trials, in which the triangle test method was used. On each trial, participants were given three glasses with identical food colouring (red, yellow, green or colourless). Two of the three glasses contained the same flavour, while one contained a different flavour (e.g. two solutions were cherry flavoured and one was strawberry flavoured). The task of the participants was to pick out the one with the different flavour. The combinations of flavours and colours are given in Table 1. They were presented to the participants in two different pseudo-randomised trial series.

**Table 1.** Combinations of Flavours and Food Colouring in Each of the 24 Trials in the Experiment.

	Flavour in			
Food colouring	Glass 1	Glass 2	Glass 3	Condition
red	strawberry	**cherry**	strawberry	congruent
red	**cherry**	strawberry	strawberry	congruent
red	**strawberry**	cherry	cherry	congruent
red	cherry	**strawberry**	cherry	congruent
yellow	grapefruit	grapefruit	**lemon**	congruent
yellow	lemon	**grapefruit**	lemon	congruent
yellow	**lemon**	grapefruit	grapefruit	congruent
yellow	**grapefruit**	lemon	lemon	congruent
yellow	cherry	cherry	**strawberry**	incongruent
yellow	cherry	**strawberry**	cherry	incongruent
yellow	**cherry**	strawberry	strawberry	incongruent
yellow	strawberry	strawberry	**cherry**	incongruent
green	**grapefruit**	lemon	lemon	incongruent
green	grapefruit	**lemon**	grapefruit	incongruent
green	lemon	lemon	**grapefruit**	incongruent
green	grapefruit	grapefruit	**lemon**	incongruent
no colour	cherry	cherry	**strawberry**	colourless
no colour	strawberry	strawberry	**cherry**	colourless
no colour	**strawberry**	cherry	cherry	colourless
no colour	strawberry	**cherry**	strawberry	colourless
no colour	**lemon**	grapefruit	grapefruit	colourless
no colour	lemon	lemon	**grapefruit**	colourless
no colour	**grapefruit**	lemon	lemon	colourless
no colour	grapefruit	**lemon**	grapefruit	colourless

*Note*. The order in which the combinations were presented was pseudo-randomised over all subjects. Correct responses in each trial are depicted in bold font.

Participants were asked to detect the differently flavoured solution by drinking the beverages one after the other from left to right. Between each glass, a break of 10 seconds was given. After the third glass was emptied, the subjects marked their response in a table provided on paper. The response time was measured from the moment the third glass was emptied until the subject’s response was marked on the sheet of paper. Subjects in the AST group were asked to additionally perform the AST during each trial in the following manner: After the first glass was emptied, the subjects continuously repeated the word ‘das’ (German word for ‘the’) aloud during the 10-second break until the second glass had to be drunk. The experimenter indicated when the 10-second break was over and the contents of the second glass could be drunk. After the second glass was emptied, the subject was again asked to repeat the AST over the next 10-second break until he or she began to drink from the third glass. After the third glass was emptied, the subject was again asked to perform the AST until he or she marked his or her response on the response sheet. After a break of at least 30 seconds, the next trial started. Participants were given a glass of colourless water from which they could drink during the inter-trial interval.

### Data Analysis

For each participant, the mean proportion of errors and the mean response times in seconds were calculated for each colour condition (congruent, incongruent and colourless). Participants could achieve a minimal number of errors of 0 and a maximal number of errors of 8 for each condition, with a maximal number of errors of 24 over all three conditions. Proportions of errors were arcsine transformed, and response times were logarithmically transformed for parametric statistical analysis. Repeated-measures analyses of variance (ANOVAs) were used to test for main effects of colour condition and group (AST vs. non-AST), as well as for interactions between the two factors. For the three-level factor colour condition, there is a risk of violation of sphericity. To account for this, the Huynh–Feldt correction factor ɛ for the degrees of freedom, as given by the Mauchly (1940) test on sphericity, will be stated where appropriate. For effect sizes, partial eta squared is reported and additionally Cohen’s *d* where appropriate.

## Results

### Error Rates

**Figure 2.**
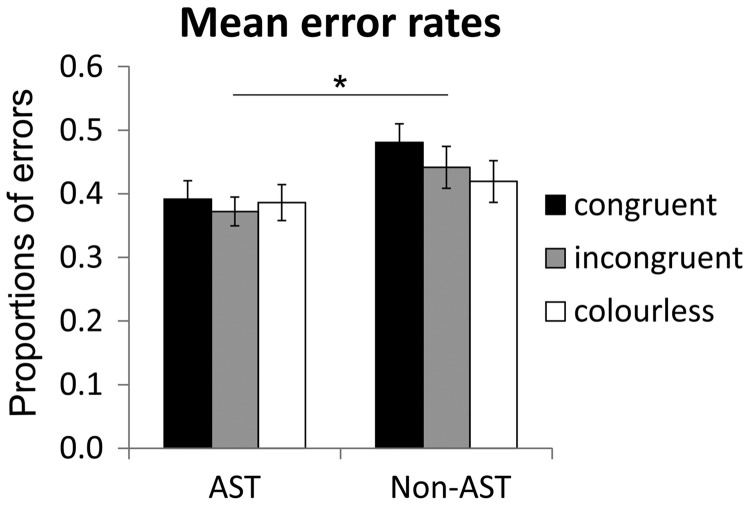
Mean proportions of errors in the gustatory discrimination task, left for the group with AST and right for the group without the task (non-AST). The AST group made significantly less errors than the non-AST group. The different colour conditions (congruent in black, incongruent in grey and colourless in white) did not significantly influence the proportions of errors. (*p < .05; error bars depict *SE*). AST =  articulatory suppression task.

Mean proportions of errors (including *SE*s) are depicted in Figure 2 for the three colour conditions congruent, incongruent and colourless and the two experimental groups with AST and without AST. Overall error rates were relatively high; however, *t* tests of number of errors in each condition against chance level yielded highly significant results (all *p*s < .001), indicating that subjects still performed clearly above chance level in all tested conditions.

A repeated-measures ANOVA (univariate approach) with the within-subjects factor colour condition (congruent, incongruent and colourless) and the between-subjects factor group (AST vs. non-AST) was performed on the arcsine transformed proportions of errors. It revealed a significant main effect of group, *F*(1, 88) = 5.30; *p* = .024; *d* = −.48; $\eta_{\text{p}}^{\text{2}}$ = .057, with the AST group exhibiting significantly fewer errors than the non-AST group. The main effect of colour condition, *F*(2, 176) = 1.01; *p* = .36; $\eta_{\text{p}}^{\text{2}}$ = .011; ɛ = 1.0, and the interaction between colour condition and group, *F*(2, 176) = .50; *p* = .61; $\eta_{\text{p}}^{\text{2}}$ = .006; ɛ = 1.0, on error rate were not significant. Pairwise comparisons between the three colour conditions (Bonferroni corrected for multiple comparisons), performed separately for each group, revealed no significant differences (all *p*s > .05).

Adding the factor flavour pair (strawberry–cherry vs. grapefruit–lemon) to the analysis revealed a significant main effect of flavour pair, *F*(1, 88) = 7.0; *p* = .01; *d* = .35; $\eta_{\text{p}}^{\text{2}}$ = .074, with significantly more errors in the strawberry–cherry flavour pair. Consequently, repeated-measures ANOVAs as stated earlier were conducted separately for the two flavour pairs. For the strawberry–cherry flavour pair, this analysis revealed a marginally significant main effect of colour condition, *F*(2, 176) = 2.38; *p* = .096; $\eta_{\text{p}}^{\text{2}}$ = .026; ɛ = 1.0, and a significant interaction between colour condition and group, *F*(2,176) = 3.27; *p* = .04; $\eta_{\text{p}}^{\text{2}}$ = .036; ɛ = 1.0. The congruent condition tended to produce more errors than the incongruent and colourless condition, especially in the non-AST group. The main effect of group was, however, not significant, *F*(1, 88) = .003; *p* = .96; *d* = .01; $\eta_{\text{p}}^{\text{2}}$ < .001. For the grapefruit–lemon flavour pair, the ANOVA yielded no significant effects, though there is a trend to overall fewer errors in the group with AST. These results reveal that the effects of colour condition were mainly driven by the strawberry–cherry flavour pair. Table 2 shows mean error rates and their respective standard errors separately for the two flavour pairs.

**Table 2.** Mean Error Rates and SEs for the Three Colour Conditions Congruent, Incongruent and Colourless, Separately for the Two Flavour Pairs and the Two Groups With and Without AST.

Flavour pair	Strawberry–Cherry				Grapefruit–Lemon			
	AST		Non-AST		AST		Non-AST	
Colour condition	Mean	*SE*	Mean	*SE*	Mean	*SE*	Mean	*SE*
Congruent	.46	.035	.53	.040	.32	.042	.44	.039
Incongruent	.40	.037	.44	.042	.34	.032	.44	.045
Colourless	.43	.041	.45	.041	.34	.037	.40	.041

*Note*. AST = articulatory suppression task; *SE* = standard error.

### Response Times

**Figure 3.**
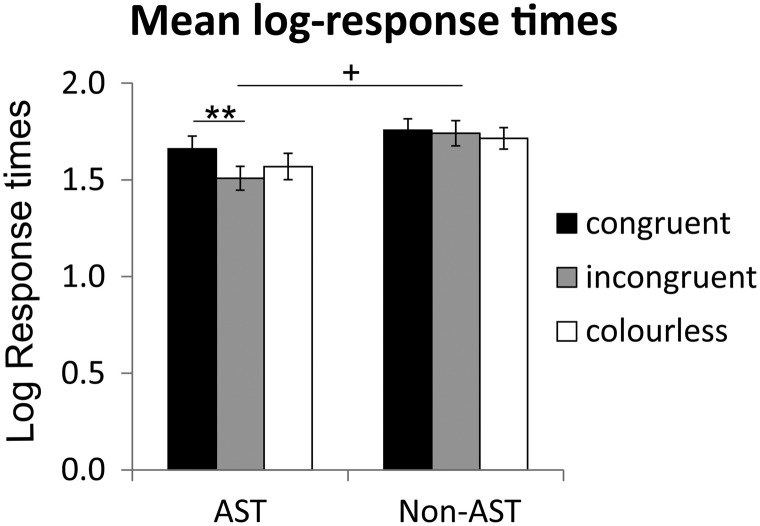
Mean log response times in the gustatory discrimination task, left for the group with AST and right for the group without the task (non-AST). The AST group responded marginally significantly faster than the group without AST. Within the AST group, participants responded significantly faster in the incongruent colour condition than in the congruent colour condition. (+*p* < .1; **p < .01; error bars depict *SE*). AST =  articulatory suppression task.

Mean logarithmically transformed response times (including *SE*s) for the three colour conditions congruent, incongruent and colourless and the two experimental groups AST and non-AST are depicted in Figure 3.

Again, a repeated-measures ANOVA (univariate approach) with the within-subjects factor colour condition (congruent, incongruent and colourless) and the between-subjects factor group (AST vs. non-AST) was performed on the log-transformed response times. The ANOVA revealed a marginally significant effect of group, *F*(1, 88) = 3.65; *p* = .059, *d* = −.40; $\eta_{\text{p}}^{\text{2}}$ = .04, indicating that the group with AST responded overall faster than the non-AST group. Also, the main effect of colour condition, *F*(2, 176) = 5.22; *p* = .006, $\eta_{\text{p}}^{\text{2}}$ = .056; ɛ = 1.0, and the interaction between colour condition and group, *F*(2, 176) = 3.17; *p* = .045, $\eta_{\text{p}}^{\text{2}}$ = .035; ɛ = 1.0, were significant. Pairwise comparisons between the three colour conditions (Bonferroni corrected for multiple comparisons), separately for the two groups AST and non-AST, revealed that in the AST group response times in the congruent condition were significantly higher than in the incongruent condition (*p* = .004). All other pairwise comparisons (Bonferroni corrected) in the AST and non-AST group, respectively, revealed no significant differences (all *p*s > .05).

Again, the factor flavour pair (strawberry–cherry vs. grapefruit–lemon) was added to the analysis. This repeated-measures ANOVA yielded no significant main effect of flavour pair, *F*(1, 88) = 2.34; *p* = .13; *d* = .09; $\eta_{\text{p}}^{\text{2}}$ = .026, but a marginally significant interaction between flavour pair and colour condition, *F*(2, 176) = 3.0; *p* = .052; $\eta_{\text{p}}^{\text{2}}$ = .033; ɛ = 1.0, and a significant three-way interaction between flavour pair, colour condition and group, *F*(2, 176) = 5.75; *p* = .004; $\eta_{\text{p}}^{\text{2}}$ = .061; ɛ = 1.0. Table 3 shows mean log response times and their respective standard errors separately for the two flavour pairs. The increased response times for the congruent condition in the AST group were mainly associated with the strawberry–cherry flavour pair.

**Table 3.** Mean Log Response Times and SEs for the Three Colour Conditions Congruent, Incongruent and Colourless, Separately for the Two Flavour Pairs and the Two Groups With and Without AST.

Flavour pair	Strawberry–Cherry				Grapefruit–Lemon			
	AST		Non-AST		AST		Non-AST	
Colour condition	Mean	*SE*	Mean	*SE*	Mean	*SE*	Mean	*SE*
Congruent	1.72	.080	1.74	.061	1.56	.063	1.74	.068
Incongruent	1.55	.062	1.72	.072	1.43	.072	1.70	.070
Colourless	1.49	.077	1.72	.062	1.61	.070	1.67	.059

*Note*. AST = articulatory suppression task; *SE* = standard error.

## Discussion

In our experiment, we aimed to replicate the study of Stevenson and Oaten (2008) in the flavour domain and to investigate the effects further by additionally analysing response times. Overall, we could observe that the application of the AST clearly improved performance (error rates and response times). The effect sizes for this result lie in a medium range (Cohen’s *d* =−.48 for error rates and *d* = −.40 for response times). This finding is to a certain extent in line with the results of Stevenson and Oaten in their ‘colour alerting’ condition. Stevenson and Oaten had their participants randomly assigned to either an ‘alerting’ condition or an ‘incidental’ condition. In the ‘alerting’ condition, participants were alerted to the fact that the odour solutions were coloured by letting them tick a box, whenever they encountered a coloured stimulus. In the ‘incidental’ condition, colour was not mentioned at all to the participants. In our experiment, participants were not explicitly alerted to the colours, but the colours of the solutions were clearly visible, and the colours of liquids that were drunk can probably be less easily ignored than the colours of solutions that were just smelled. Therefore, we would argue that our procedure was probably more similar to Stevenson and Oaten’s ‘alerting’ condition than to their ‘incidental’ condition.

With regard to the different colour conditions, Stevenson and Oaten (2008) found that the AST improved discrimination especially in the incongruent colour condition, where their participants in the non-AST group exhibited significantly more errors than in the congruent and colourless conditions. Interestingly and surprisingly, we could not find a similar effect. Error rates did not differ significantly between the three colour conditions, neither in the AST group nor in the non-AST group. Especially, the result for the non-AST group is surprising, since it was expected that, if colour assists identification via an internal verbal labelling process, this should enhance discrimination in the congruent condition, whereas it should hamper it in the incongruent condition. In contrast, the trend (albeit not significant) goes in a different direction, with participants in our experiment performing slightly worst in the congruent colour condition. Even more surprising were our results with respect to the response times in the AST group. Normally, one would expect differences between the colour conditions to decrease during the application of the AST, which was more or less the case for the error rates. But for the response times, it was just the AST group that showed a significant difference between the congruent and the incongruent colour condition. It appears that when the colour was appropriate for both target and distractor flavours, participants had more difficulty to distinguish between them, resulting in higher response times. This effect was apparent for both, AST and non-AST groups (see Figure 3). Apparently it was easier for them to do the task, when the liquids were incongruently coloured or uncoloured. Future research should determine if this might be a sign of low-level multisensory integration in flavour perception, independently of top-down influences of colour on flavour discrimination.

Comparing the two flavour pairs strawberry/cherry and grapefruit/lemon, it becomes evident that any effect of colour condition was most pronounced in the strawberry/cherry flavour pair. This might indicate that only with this flavour pair, a true congruency/incongruency effect was observed. The choice of the colours ‘yellow’ as congruent and ‘green’ as incongruent in the grapefruit/lemon flavour pair appears to be less appropriate in hindsight, since certain citrus fruits, such as limes, are green.

A limitation of our study is that we could not present the full set of six possible orders in the triangle test for each flavour pair, because the amount of liquids subjects had to drink in the course of the experiment in that case would have exceeded a reasonable amount. With this aspect in mind, we tested for position effects within each condition and group with a one-factorial ANOVA. This control analysis yielded no significant results (all *p*s > .05), indicating that the position of the deviant sample in the triangle of each trial did not have any systematic effects on our results. Similarly, adding the factor trial series to the repeated-measures ANOVAs yielded no significant main effect for trial series, neither for performance rates nor for response times.

Another limitation of the current study is that the flavours were presented in very low concentrations, and thus, the flavour-discrimination task on a whole was rather difficult. We had mean numbers of errors between three and four (out of a total of eight trials per condition), while Stevenson and Oaten (2008) reported much lower numbers of mean discrimination errors. On the other hand, the performance of our subjects was still significantly above chance level, as stated in the Results section. Nevertheless, a floor effect could have occurred in some subjects, limiting the interpretation of our results.

Moreover, it is unclear to what extent participants in our study were aware of the meaning or aim of the different colour conditions. Future experiments might incorporate conditions that modulate possible attentional states of the participants to further explore the effects of top-down influences and attention on flavour discrimination.
